# Lgr5+ stem cells and their progeny in mouse epidermis under regimens of exogenous skin carcinogenesis, and their absence in ensuing skin tumors

**DOI:** 10.18632/oncotarget.10475

**Published:** 2016-07-07

**Authors:** Gerline C. van de Glind, Jacoba J. Out, Heggert G. Rebel, Cornelis P. Tensen, Frank R. de Gruijl

**Affiliations:** ^1^ Department of Dermatology, LUMC, Leiden, 2333RC, The Netherlands

**Keywords:** stem cells, Lgr5, lineage tracing, UV, skin carcinogenesis

## Abstract

Actively proliferating Lgr5+ skin stem cells are found deep in the hair follicle (HF). These cells renew the HF and drive its expansion in anagen phase. Their long residence and continuous mitotic activity make them prime candidates to transform into skin tumor-initiating cells. This was investigated by subjecting Lgr5-EGFP-Ires-CreERT2/R26R-LacZ mice (haired and hairless) to chemical and UV carcinogenic regimens. In the course of these regimens Lgr5+ cells (EGFP+) remained exclusively located in HFs, and in deep-seated cysts of hairless skin. In haired mice, progeny of Lgr5+ stem cells (LacZ+ after a pulse of tamoxifen) appeared in the interfollicular epidermis upon UV-induced sunburn and in TPA-induced hyperplasia. In hairless mice the progeny remained located in deep-seated cysts and in HF remnants. Progeny in hairless skin was only detected interfollicularly at a late stage, in between outgrowing tumors. Lgr5+ stem cells were absent in the ultimate tumor masses, and no tumor appeared to be a (clonal) expansion of Lgr5+ cells (52 tumors with tamoxifen at the start of carcinogenesis, 42 tumors with tamoxifen late during tumor outgrowth). In contrast to CD34/K15+ quiescent bulge stem cells, actively proliferating Lgr5+ stem cells do therefore not appear to be tumor drivers in experimental skin carcinogenesis.

## INTRODUCTION

The skin protects our body against environmental insults. In order to remain vital and functional, the outermost layer, the epidermis, constantly renews itself from dedicated adult stem cells. Classically, these stem cells were thought to be rarely dividing ‘quiescent stem cells’ [1]. However, a novel class of continuously dividing stem cells, Lgr5+, was found to drive epithelial turnover. These cells were first described for the intestine [2] and later also for the skin [3]. In the skin these Lgr5+ stem cells are located in the lower part of the bulge and in the bulb of the hair follicle (HF), depending on the hair cycle phase. These stem cells renew the cells in the HF [3]. Upon wounding progeny of the Lgr5+ stem cells migrate to the interfollicular epidermis (IFE), to replace damaged cells [4]. Lgr5 is a Leucine-rich repeat-containing G-protein-coupled receptor located on the cell membrane. Upon binding R-spondin Lgr5 enhances Wnt-signaling, with the *Lgr5* gene as one of the transcriptional targets [3].

By their very nature adult stem cells are long residing and divide to renew surrounding tissue. Consequently they are expected to run an increased risk of accumulating mutations, and thus become tumor stem cells or tumor initiating cells [5, 6]. We were therefore interested in how Lgr5+ stem cells react to exogenic carcinogenic stimuli and whether they become initiating cells of skin carcinogenesis.

In studies on skin carcinogenesis mainly two experimental models are used. In one established model hairless mice are chronically UV-exposed to induce squamous cell carcinomas (SCCs). These tumors carry typical UV-signature mutations in *p53* [7]. In the other classical ‘two-stage’ model tumors are initiated in the shaven skin of haired mice by a single application of a genotoxic agent, e.g., 7,12-Dimethylbenz[a]anthracene (DMBA). Tumor development is subsequently stimulated by repeated applications of a ‘tumor promoter’, a hyperplasia inducing irritant. Originally croton oil was used as a tumor-promoter, and later on its active ingredient, 12-*O*-Tetradecanoylphorbol-13-acetate (TPA) [8]. Besides hyperplasia of the IFE, TPA induces anagen in HFs of haired mice [9, 10], which would implicate Lgr5+ stem cells.

Using the second model, mostly frank papillomas with *H-Ras* mutations develop [11, 12], and more rarely SCCs. We showed that the UV-induced SCCs originate from the IFE [13]. Whereas an earlier study showed that chemically induced SCCs originate from the HFs [14]. Chemically induced skin tumors contain CD34+ stem cells [15]. These cells are normally located in the bulge of HFs in haired mice (but not in hairless mice) [16]. The development of chemically induced skin tumors is impaired in CD34-null epidermis [17]. But despite the absence of CD34+ bulge cells, hairless mice are susceptible to chemically induced skin tumors [18]. In this study we investigated Lgr5+ stem cells and their progeny (“Lgr5 progeny” for short) under exogenous carcinogenic stimuli. We aimed to establish whether these cells drive the outgrowth of skin tumors. To this end, we used Lgr5-EGFP-Ires-CreERT2 mice carrying a Rosa26-LacZ reporter in a haired as well as in a hairless background. We studied Lgr5+ cells (EGFP+) and their progeny (LacZ+) in skin samples (cross sections, whole mounts and epidermal sheets): a) taken after a single UV overexposure, with massive apoptosis in the epidermal basal layer while overlying epidermis remained intact (i.e. no wounding), b) from sub-acute chronic UV and chemically induced epidermal hyperplasia and c) from UV and chemically induced tumors. A schematic overview including the time line of the experiments can be found in Supplementary Figure S1.

## RESULTS

### Lgr5 expressed in skin of hairless mice

Adult hairless SKH-1 mice do not have normal cycling HFs; their follicles seem to be frozen in catagen. The hair follicle remnants are connected by a hardly discernible string of cells to deep-seated cysts (putative outgrowths of bulbs) [13]. Given these abnormal HFs, we were interested if hairless mice expressed Lgr5. In haired mice Lgr5 is normally expressed in the bulge and bulb regions of HFs (see Supplementary Figure S2). In hairless mice we found EGFP-expressing Lgr5+ stem cells in the deep-seated cysts where, after tamoxifen-induced activation of Cre, their LacZ+ progeny built up (see Supplementary Figure S2). Furthermore, we observed some Lgr5+ cells and progeny cells higher up at the bottom of the hair follicle remnant (just below sebaceous glands), and in the string of cells running down to the cyst (Supplementary Figure S2D).

### Lgr5+ stem cell progeny contributed to the repopulation of an ablated interfollicular basal layer in haired mice

**Figure 1:**
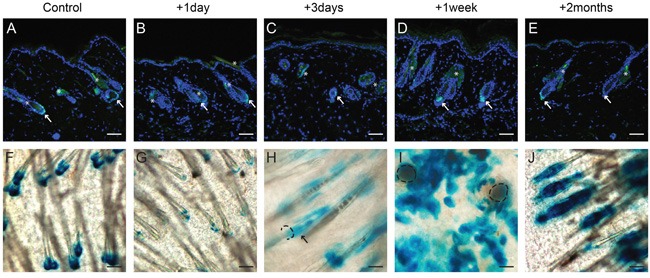
Lgr5+ stem cells remain in their homeostatic location, but Lgr5 progeny repopulated the epidermal basal layer after UV overexposure in haired mice Paraffin sections of control mice and mice that received an UV overexposure were stained with an anti-EGFP antibody **A-E**; whole mounts **F-J** were stained for LacZ expression (representative pictures are shown). A+F show Lgr5+ stem cells and their progeny in control mice without any UV exposure (homeostasis). B-E show the location of Lgr5+ stem cells at different time points after UV overexposure; no differences were observed compared to the control mice (see arrows). G-J show the Lgr5 progeny at different time points after overexposure. One week after overexposure progeny clearly migrated out of the hair follicle into the epidermal basal layer (I), this was not observed in control mice. Hair follicle orifice (in H+I) contoured; and arrow in H points at rim staining. scale bar = 100μm (in A-G and J), scale bar = 50μm (H+I).

We used a tolerable overdose of UV (3.6MED for haired and 5 MED for hairless mice, see Materials and Methods) to ablate the epidermal basal layer [13]. With this dose the basal layer became massively apoptotic (see Supplementary Figure S3), but the overlaying cell layers stayed intact and thus no wounds occurred. We found the Lgr5+ stem cells at successive time points after overexposure to be confined to the HFs, as in homeostasis: at the bottom (bulge or bulb regions) of HFs in haired mice (Figure 1). In hairless mice Lgr5+ cells remained in the cysts and higher up at the bottom of hair follicle remnants (see Supplementary Figure S4 and data not shown). To trace Lgr5 progeny, tamoxifen was administered prior to UV overexposure. The progeny of Lgr5+ stem cells in hairless mice remained restricted to the cysts and hair follicle remnants (see Supplementary Figure S4 and data not shown). However, in haired mice we observed Lgr5 progeny in the IFE: after 3 days progeny was limited to the rims of hair follicles and then clearly migrated into the IFE by 1 week after overexposure (see Figure 1I). But after two months progeny was almost completely lost from the IFE. In the controls without an ablating UV dose we did not find any Lgr5 progeny in the IFE (Figure 1F and Supplementary Figure S4E).

### Progeny of Lgr5+ stem cells migrated into the IFE after chemically induced hyperplasia

**Figure 2:**
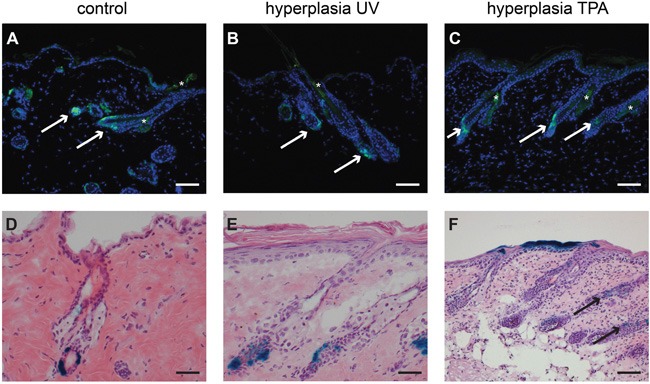
Lgr5 progeny migrates out of the hair follicle into the IFE after hyperplasia induced by TPA in haired mice Paraffin sections of haired mice were stained with an anti-EGFP antibody **A-C** and frozen sections **D-F** were stained for LacZ expression (representative pictures are shown). B and C show the location of EGFP-expressing Lgr5+ stem cells after hyperplasia induced by UV (B) or chemically by TPA (C), the location is the same as in untreated control mice (A). After UV induced hyperplasia (E) the Lgr5 progeny was found in the same location as in de control mice (D). However, after TPA induced hyperplasia Lgr5 progeny migrated out of the hair follicle into the epidermal basal layer (F). scale bar = 100μm (in A-C and F), scale bar = 50μm (D+E).

Epidermal hyperplasia was induced in haired and hairless mice either by chronic subacute UV exposure or by TPA applications (see Materials and Methods). Tamoxifen was administered prior to hyperplasia inducing regimen. The Lgr5+ stem cells remained in their homeostatic location in haired and hairless mice (see Figure 2B+2C for haired and Supplementary Figure S4C+S4D for hairless mice). However, in haired mice the progeny of Lgr5+ stem cells migrated into the IFE in TPA-induced hyperplasia (see Figure 2F). This was not the case in UV-induced hyperplasia (Figure 2E), nor in hyperplasia in hairless mice up to 8 weeks of TPA or UV exposure (see Supplementary Figure S4G+S4H).

### Interfollicular inter-tumoral Lgr5 progeny was present in both haired and hairless mice

**Figure 3:**
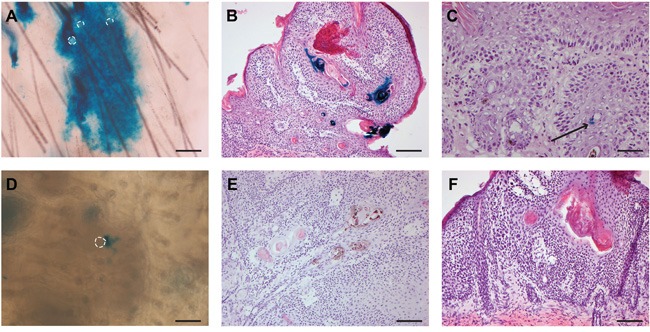
Progeny of Lgr5+ stem cells is (largely) absent in skin tumors; in hairless mice progeny of Lgr5+ stem cells migrated into the IFE only after prolonged treatment (over 6 months) with carcinogenic stimuli Whole mount skin samples of haired mice after chemocarcinogenesis **A** and of hairless mice after UV-carcinogenesis **D** were stained for LacZ and showed interfollicular Lgr5 stem cell progeny. Hair follicle orifices are contoured (dotted lines). Tumors were induced by chemocarcinogenesis **B+C** in haired mice or by UV exposure **E+F** in hairless mice, respectively. LacZ lineage tracing was induced at the start of the experiment B+E or when tumors were formed (C+F), and tumor sections were stained for LacZ expression. Sparse inclusions of Lgr5 progeny in terminally differentiated parts were only observed in haired mice after chemocarcinogenesis when tracing was induced at the start of the experiment (B, some positivity seen near and in keratin pearls in 7/29 tumors). Scale bar in D = 50 μm, all other scale bars represent 100 μm.

We also investigated the uninvolved hyperplastic skin adjacent to tumors. In the haired mice subjected to chemocarcinogenesis (TPA treatment for >6 months), we observed interfollicular Lgr5 progeny similar to what we found earlier in the chemically induced hyperplasia experiments (Figure 3A). Surprisingly, we detected interfollicular Lgr5 progeny in hairless mice as well at this stage between both chemo (Supplementary Figure S5) and UV tumors (Figure 3D).

### Lgr5+ stem cells were not the tumor-driving cells in skin tumors

Our main interest in this study was to investigate the role of Lgr5+ stem cells in tumor formation in the skin. Therefore we subjected mice either to UV-induced skin carcinogenesis (only in hairless mice; see Materials and Methods) or chemically induced carcinogenesis using DMBA and TPA (both in hairless and haired mice); see Supplementary Table S2. Each group was divided into two; one group received tamoxifen injections to initiate lineage tracing at the beginning of the experiments and the other group received the injections when tumors (> 4mm) had formed. Giving the injections at the beginning of the experiments enabled us to study clonal expansion of the Lgr5 progeny into the tumor. If so, the progeny should make up the complete tumor mass, resulting in entirely blue tumors after staining for LacZ. Injecting after the first tumors had occurred and subsequent tracing for 2-3 weeks would reveal whether Lgr5+ stem cells in the tumor were fueling the growth.

Under the chemocarcinogenic regimen mainly (exophytically growing) papillomas developed in contrast to UV carcinogenesis where mainly (endophytically growing) SCCs developed. However, independent of tumor type we did not find any EGFP-positive cells in the tumor masses (n=32 tumors). Most Lgr5+ stem cells that we observed were located in hair follicle-like structures below or neighboring the tumor mass and none in the tumor mass itself (data not shown). Correspondingly, progeny did not significantly contribute to the tumor mass; some sporadic minor LacZ+ patches in terminally differentiated parts of the tumors were most likely inclusions. In the haired mice subjected to chemocarcinogenesis and traced from the beginning of the experiment, we detected some rare LacZ+ keratin ‘pearls’ and sparse LacZ+ cells in the most differentiated region of the tumor (16 out of 29; see Figure 3B). Progeny was clearly present in neighboring HFs, but the bulk of the tumor mass was negative. In the group where the tracing was initiated when tumors had already formed, almost no Lgr5 progeny was found at all in the tumors (n=11, see Figure 3C). And again we did observe progeny in neighboring HFs. Tumors that developed in hairless mice did not show substantial Lgr5 stem cell progeny either, irrespective of the carcinogenic regimen (n= 54, see Figure 3E+3F and Supplementary Figure S5).

A lack of Lgr5 expression in tumors could be due to promoter hypermethylation. Previously, LGR5 promoter hypermethylation was shown in colorectal cancer [19]. We therefore checked our skin tumors for hypermethylation of the relevant promoters, i.e of *Lgr5* and *Rosa*. We found no indication of hypermethylation of these promoters (see Supplementary Figure S6 for the methylation-specific melting curves and Supplementary Table S1 for used primers).

### Stem cell markers CD34 and Sox2 are expressed in UV-induced skin tumors

Stem cell markers, CD34 and Sox2, are known to be expressed in chemically induced skin tumors. We were interested whether these markers were also expressed in UV-induced tumors. CD34 is expressed in the bulge region of the HF but is not expressed in the hair follicle remnants of hairless mice [16], while Sox 2 is expressed in the dermal papilla [20].

**Figure 4:**
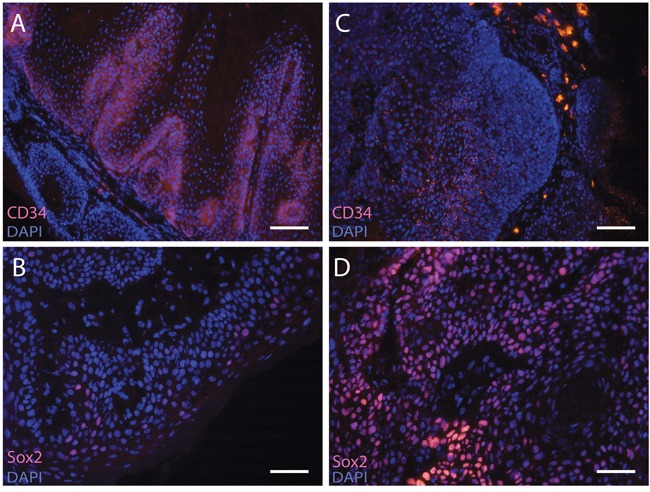
CD34 and Sox2 expression in skin tumors Sections of tumors were stained with anti-CD34 **A+C** or anti-Sox2 **B+D**. Both chemically induced tumors in haired mice (A+B) (CD34+ 15 of 21 tumors and Sox2+ 7 of 8 tumors) as well as UV-induced tumors in hairless mice (C+D) showed CD34 (6 of 14 tumors) and Sox2 expression (10 of 11 tumors). The CD34 expression in hairless mice was located in the differentiated compartments and more granular (C) compared to the haired mice (A). Scale bar = 100μm.

As depicted in Figure 4, CD34 was expressed in the proliferative compartment of chemically induced tumors in haired mice (15/21 tumors positive, Figure 4A). Surprisingly, the tumors induced in hairless mice also showed CD34 positivity but in the differentiated compartments and more granularly distributed than in the haired mice (Figure 4C) (8/10 positive of chemically induced tumors, data not shown; and 6/14 of UV-induced tumors). Sox2+ cells were found in chemically and UV-induced tumors from both haired and hairless mice (Figure 4B+4D; haired chem. 7/8 positive; hairless chem. 8/10 and hairless UV 10/11). However, Sox2 expression was apparently not restricted to stem cells as it was also observed in differentiated compartments of tumors (Figure 4B).

## DISCUSSION

In this study we showed that Lgr5+ stem cells remain in the hair follicle upon challenges with carcinogenic agents, but their progeny could end up in the IFE. The Lgr5+ stem cells did not appear to become tumor-initiating cells in skin carcinogenesis by exogenous agents. With lineage tracing from the start of the skin carcinogenic regimen no tumors showed up completely stained blue from LacZ activity, which would have indicated a (clonal) expansion of Lgr5 progeny. A schematic depiction of the locations of Lgr5+ stem cells and their progeny in the different experiments is given in Supplementary Figure S7.

Overall, the Lgr5+ stem cells were mainly located at the bottom of the cycling HFs in haired mice: in the lower bulge region in telogen and in the bulb in anagen. As shown by Jaks et al [3] the Lgr5+ cells only partially overlapped with CD34+ bulge cells in telogen, but not in anagen when Lgr5+ cells were present in the bulb. We infer that the Lgr5+ cells are more bulb-related than bulge-related stem cells as they migrate up and down in the hair cycle at the bottom of the HF. Furthermore, we are the first to show that hairless mice, lacking normal cycling HFs and CD34+ bulge cells, do harbor Lgr5+ stem cells in their skin. These cells and their progeny are located in cysts that appear to be expanded bulb remnants. At the point where a string of cells connected a cyst to a hair follicle remnant (just below the sebaceous glands) we also observed some Lgr5 expressing cells (see Supplementary Figure 4B and [13]).

In haired mice the progeny of Lgr5+ stem cells migrated to the interfollicular epidermis after UV-induced ablation of the epidermal basal layer and during chemically driven epidermal hyperplasia, but not during UV-driven hyperplasia up to 8 weeks. In the hairless mice however, no Lgr5 progeny appeared in the IFE in these experiments. Apparently, the location and activity of the Lgr5+ stem cells in haired mice, enables these cells to contribute to the IFE if adequately stimulated. TPA can enter the orifices of HFs and easily reach the Lgr5+ stem cells. This also explains the strong follicular hyperplasia, not observed with sub-acute UV exposure. UV radiation cannot penetrate deeply into the HF thus leaving the Lgr5+ stem cells unaffected. In hairless mice most of the Lgr5+ cells reside in the deep-seated cysts which make them poorly accessible for both TPA and UV. The effect of UV on Lgr5+ cells is most likely indirect, stemming from stress factors released higher up in the skin (most strongly after a severe sunburn). Not only are the deep-seated Lgr5+ cells inaccessible, but their progeny has to pass through a narrow corridor over a large distance in order to reach the surface and migrate into the IFE. This combination of factors may explain why Lgr5 progeny emerged in the IFE of hairless mice only after prolonged carcinogenic stress, i.e., at a very late stage in between the tumors (see Figure 3D and Supplementary Figure S5A).

Previously, Kasper et al described that after incisional wounding, involving the dermis, the Lgr5 progeny contributed to the repair of the epidermis [4]. They suggested that severe damage (including a strong or long-lasting inflammatory response) is necessary to induce Lgr5 progeny to migrate into the IFE [4]. Here we show that loss of (only) the epidermal basal layer (in combination with an inflammatory skin reaction) appears to be a sufficient condition for Lgr5 progeny to migrate to the IFE to contribute to its repair.

In the tumors we did not observe any substantial clonal expansion of Lgr5+ stem cells. Instead sparse remnants of progeny were found, most likely inclusions of wild type cells. We reasoned that our results might have been affected by methylation of CpG islands in the *Lgr5* or *Rosa* promoter. But we found no proof of methylation (see Supplementary Figure S6). Furthermore, we detected LacZ staining in nearly all of our samples in adjacent skin tissue. This served as an internal positive control, demonstrating that progeny was properly detected.

Kasper et al [4] found Lgr5 stem cell progeny in interfollicular foci of basaloid hyperproliferation. They induced these lesions by activating the Hedgehog pathway in basal cells (K5tTA/TREGLI1) and by wounding. Without wounding and subsequent recruitment of Lgr5 progeny to the IFE, no Lgr5 progeny was found in lesions in the IFE, only in those associated with HFs. Progeny of Lgr5+ stem cells was observed in BCC-like tumors when these tumors were initiated from Lgr5+ stem cells (Lgr5-creER^T2^/Ptch^fl/fl^) [4]. Da Silva-Diz et al. found Lgr5 progeny in papillomas induced by HPV proteins expressed in basal cells [21]. In our chemocarcinogenesis experiments Lgr5 progeny was recruited to the IFE (see Figure 2F), but did not contribute to any significant extent to the ultimate tumor masses.

Liu et al found up-regulation of LGR5 using Western Blots of human SCCs [22]. They used a polyclonal antibody that might have cross reacted with other (LGR) proteins, and they did not confirm their results with qPCR [22]. Furthermore, studies on the gene expression profiles of human SCCs have found no indication for the enrichment of LGR5-expressing cells [23, 24]. Hence, combined with our results we conclude that Lgr5 stem cells do not become tumor-initiating cells of cutaneous SCCs from exogenous carcinogens.

As reported earlier, we found CD34+ cells in the proliferative compartment (bordering the stroma) of chemically induced skin tumors of haired mice. These cells were shown to display the features of tumor-initiating cells [15, 17], and in normal HFs they include the classical quiescent stem cells [1]. Hence, the CD34+ bulge cells appear to be targeted in two-stage chemocarcinogenesis to become tumor-initiating cells. Actually, K15+ bulge cells (overlapping CD34+ cells) were shown by lineage tracing to drive the growth of chemically induced skin tumors [15].

Unexpectedly, we did find CD34 expression in the skin tumors from hairless mice, albeit aberrantly with a granular pattern in the differentiated cells; evidently not marking any tumor stem cells. Another marker expressed in various stem cells, transcription factor Sox2, was also found to be a driver of skin tumors [25]. In our experiments these cells were present to variable degrees in tumors induced chemically and by UV irradiation in hairless and haired mice; sometimes mainly in differentiating cells, clearly not marking stem cells (see Figure 4B).

We conclude that the novel class of continuously proliferating stem cells bearing the Lgr5 membrane receptor does not entail tumor-initiating cells in experimental skin carcinogenesis by exogenous agents (DMBA/TPA and UV radiation). In contrast, it has been established earlier that CD34+ quiescent stem cells in the bulge are targets for transformation into tumor-initiating cells in chemocarcinogenesis [15, 17]. We have recently found indication that interfollicular quiescent stem cells may become initiating cells of persistently growing UV-induced skin tumors [26]. These results suggest that the quiescent stem cells are more vulnerable to cancerous transformation than the actively cycling Lgr5+ stem cells. The reason of this discrepancy between these two types of stem cells could be a difference in DNA repair capacity and apoptotic response, where DNA damage has been shown to accumulate and be retained over long periods of time in quiescent stem cells [14, 27].

## MATERIALS AND METHODS

### Mice

Lgr5-EGFP-Ires-CreERT2 and RosaSOR-LacZ mice (Jackson Laboratories, Bar Harbor, USA) were crossed to incorporate the LacZ reporter for lineage tracing upon administering tamoxifen [3]. These Lgr5-EGFP-Ires-CreERT2/R26R-LacZ mice were also backcrossed into a hairless background using Crl:SKH1-HR hairless mice (Charles River, Sulzfeld, Germany).

Both male and female mice entered the experiments at 6-10 weeks of age (for each time point in the hyperplasia and ablation experiments n=4). They were kept individually in Macrolon type 1 cages at 25 ± 2°C and about 50% humidity in a 12 hours light-12 hours dark cycle during experiments. The room in which the mice were kept and experiments were performed was illuminated by fluorescent tubes that did not emit any UV radiation. Standard chow and tap water were available ad libitum.

As legally required, all mouse experiments were performed with the approval of the Leiden University Medical Centers' ethics committee for animal experiments (approval number DEC 10229) and executed according to EU regulations on animal experiments (Directive 2010/63/EU).

### Experimental outline

A schematic overview of the experiments including time points of administering tamoxifen and of taking samples is presented in Supplementary Figure S1.

### Cre activation by tamoxifen

Mice received three i.p. injections of tamoxifen (5mg/injection, T5648 Sigma-Aldrich, Zwijndrecht, The Netherlands) over three days to activate lineage tracing (LacZ-expressing cells) immediately prior to the start of the UV overexposure and hyperplasia experiments (see below). In the tumorigenesis experiments mice were divided into two groups: one group received tamoxifen injections at the start of the experiment and the other group received tamoxifen injections when two tumors ≥4 mm developed. The mice in this last group were sacrificed 2-3 weeks after activation of lineage tracing.

### UV radiation

Philips TL-12/40W tubes were mounted over the cages and switched on and off automatically to deliver intended doses (output of 54% in UV-B, 280-315 nm, and 46% in UV-A, 315-400 nm). Under these lamps the minimal edema/erythemal dose (MED) was determined to be 900 and 500 J/m^2^ UV for haired and hairless mice, respectively. To induce hyperplasia, mice were irradiated daily with 1 MED for 4-8 weeks. This same dose was used in the UV carcinogenesis experiments, where mice were daily irradiated until they had at least two ≥4 mm tumors. UV carcinogenesis was only performed with hairless mice (n= 10, 5 early induction of lineage tracing and 5 late induction) as shaven haired mice (C57BL6) in our laboratory started wounding themselves by severe scratching after months of chronically UV exposure, before developing any skin tumors [28].

For the overexposure experiments we used a higher dose that was just tolerable (no wounds) but largely ablated the basal layer of the epidermis (for haired mice 3.6 MED and for hairless mice 5 MED; note that this amounts to approximately 3 kJ/m^2^ UV for both mouse strains).

### DMBA and TPA applications

For the chemocarcinogenesis experiments mice (haired: n=5 early induction of lineage tracing and n=5 late induction; hairless n=4 early induction and n=4 late induction) received a DMBA application (100μg, 7,12-Dimethylbenz[a]anthracene, D3254, Sigma-Aldrich), on day 1. From day 8 onward they received TPA treatment (12-O-tetradecanoylphorbol-13-acetate, P8139, Sigma-Aldrich) twice a week until at least two ≥4 mm tumors developed. With each application, 10μg TPA in acetone was applied on approximately 6 cm^2^ of dorsal skin using a fine brush. Haired mice were priorly shaven to remove hair covering the dorsal skin. For the chemically induced hyperplasia experiments mice received TPA applications twice a week for 6 weeks.

### Tissue preparation

Mice were sacrificed by CO_2_ asphyxiation. Dorsal and ventral skin was isolated and prepared using different methods. Samples for the Caspase-3, β-galactosidase, CD34 and Sox2 stainings were embedded in Tissue-tek, snap frozen in liquid nitrogen and stored at −80°C until sectioning and staining. Samples for the anti-EGFP staining were fixed overnight in PBS-buffered 4% formaldehyde solution (Addedpharma, Oss, The Netherlands) and embedded in paraffin. Whole mount biopsies were cut into pieces of 5×5 mm and incubated in 20 mM EDTA (Baker, Deventer, The Netherlands) in PBS O/N at 37°C. The next day, they were washed with PBS, fixed in PBS-buffered 4% formaldehyde solution for 5 min and incubated O/N with X-gal solution (1mg/ml X-gal, 5mM ferrothiocyanide, 5 mM ferrithiocyanide, 2mM MgCl_2_ in PBS). After incubation they were embedded in Kaisers glycerin.

Tumors were either snap frozen in liquid nitrogen or fixed in PBS-buffered 4% formaldehyde solution and embedded in paraffin.

### Immunohistochemistry

#### Active caspase-3 staining

Cryosections were cut at 6μm thickness and fixed in acetone containing 0.3% H_2_O_2_. The sections were blocked with 2% Normal Human Serum (NHS) for 20 minutes. After blocking, the sections were incubated overnight at 4°C with anti-active caspase 3 (1:100, ab 2302, 3509322, Abcam, Cambridge, UK). The next day the sections were incubated for one hour with Goat-anti-Rabbit (IgG)-biotin (1:300, Vector Laboratories, Inc Burlingame, USA) followed by streptavidin (1:100, RPN1051v, GE Healthcare UK Limited) for 45 minutes. The staining was visualized with 20mg 3,3′-Diaminobenzidine (D5905, Sigma-Aldrich, Zwijndrecht, The Netherlands) in 100ml of PBS and 100μl of H_2_O_2_. Sections were counterstained with haematoxylin and mounted in Kaisers' glycerin.

#### EGFP staining

Paraffin samples were cut at 5μm thickness and incubated at 60°C O/N. The next day, they were dehydrated and antigen retrieval was performed with antigen unmasking solution (H-3300, Vector Laboratories, Inc Burlingame, USA) in a pressure cooker for 5 min. Non-specific binding was blocked with PBS/0.1% Tween for 2 h followed by incubation with anti-EGFP (1:200, ab139070, Abcam, Cambridge, UK) at 4°C O/N. The sections were incubated with secondary antibody Alexa Goat anti-chicken 488 (1: 250, Life technologies, Bleiswijk, The Netherlands) and nuclei were stained with DAPI for 5 min (1:3000, D1306, Invitrogen, Bleiswijk, The Netherlands). The sections were mounted with Vectashield mounting medium for fluorescence (H-1000, Vector Laboratories, Inc. Burlingame, USA).

#### β-galactosidase staining

Cryosections were cut at 6μm thickness and fixed with PBS-buffered 4% PFA (ROL 164810, AddedPharma, Oss, The Netherlands) for 10 min at RT. Sections were washed with PBS and with Rinse solution (2mM MgCl_2_, 0,01% NP40 in PBS) and incubated O/N at 37°C with β-galactosidase staining solution (5mM K_3_Fe(CN)_6_, 5mM K_4_Fe(CN)_6_•3H_2_O, 1mg/ml X-gal in Rinse solution). Sections were washed with rinse solution and counterstained with haematoxylin and eosin. Sections were dehydrated and embedded in Depex (18243.01, Serva Electrophoresis GmbH, Heidelberg, Germany).

#### CD34 staining

Cryosections embedded in Tissue-tek (Sakura Finetek Europe, Zoeterwoude, The Netherlands) were cut at 6μm and dried on Superfrost Plus glass slides. The sections were fixed in acetone for 10 min and blocked with 2% normal goat serum (NGS, Dakocytomation, Heverlee, Belgium) and 1% BSA in PBS. Followed by incubation of anti-CD34 antibody (1:50, purified anti-mouse-CD34 14-0341-82, eBioscience, Vienna, Austria) in 2% goat serum and 1% BSA in PBS over night at 4°C. Goat-anti-Rabbit-Cy3 antibody (1:500, 111-165-003, Jackson Immunoresearch Laboratories, Inc, West Grove, USA) in 1 % BSA in PBS was incubated for 1hr at RT. Nuclei were labelled with DAPI (1:3000, D1306, Invitrogen, Bleiswijk, The Netherlands) for 2 min and sections were embedded with Vectashield (H-1000, Vector Laboratories, Burlingame, USA).

#### Sox2 staining

Frozen sections were cut at a thickness of 6μm and fixed in paraformaldehyde in PBS for 30 min. The sections were incubated with blocking solution (1% BSA and 2% NGS in PBS) for 1hr at RT. Subsequently, the sections were incubated with Rabbit anti-Sox2 antibody (1:500, Epitomics 2683-1, Abcam Cambridge, UK) overnight at 4°C). The following steps were the same as described above for the CD34 staining. So Goat-anti-Rabbit-Cy3 was used to visualize Sox2 and DAPI to visualize the nuclei, sections were embedded using Vectashield.

Negative controls were stained without first antibody to check for background signal.

Images were acquired using a Zeiss Axioplan 2 microscope with the 10x and 20x objectives, Axiocam camera and dedicated software for immunohistochemistry. For fluorescent pictures a Leica DM 5000B Microscope was used with 5x, 10x and 20x objectives and a Leica DFC300 FX Camera with dedicated software. Final pictures were formatted in Adobe Photoshop CS6 or Adobe Illustrator CS6 and representative cases are presented in Results.

## SUPPLEMENTARY DATA TABLES AND FIGURES


