# Supplementary material for: Lgr5+ stem cells and their progeny in mouse epidermis under regimens of exogenous skin carcinogenesis, and their absence in ensuing skin tumors
Source: Oncotarget. 2016 Jul 7;7(32):52085–94. doi: 10.18632/oncotarget.10475 (PMC5239536; doi:10.18632/oncotarget.10475)
Supplement: Supplementary file 1 [file oncotarget-07-52085-s001.pdf]

Lgr5+ stem cells and their progeny in mouse epidermis under regimens of exogenous skin carcinogenesis, and their absence in ensuing skin tumors

SUPPLEMENTARY DATA

METHODS

Methylation assay

DNA was isolated from frozen tissue using the DNeasy kit (Qiagen) according to manufacturer's protocol. Bisulphite conversion of extracted DNA was performed using the EZ DNA methylation kit (Zymo Research, Orange, CA). Bisulphite primer sequences were designed to amplify the CpG island in the close proximity of the TSS of *Lgr5* and *Rosa.* Primers were developed in such a way that both methylated as well as unmethylated sequences were amplified using the same bisulphite-treated DNA as PCR template (Supplementary Table SI). Amplification was performed with iQ SYBR Green Supermix on a CFX384 Touch Real-Time PCR Detection System (Bio-Rad, Veenendaal, The Netherlands) using a touchdown PCR protocol with the following parameters: denaturing at 95°C for 30 seconds (ramp rate at 2°C per second), followed by 7 cycles of annealing at 65°C to 58°C for 40 seconds (with a 1°C decrement per cycle) and extension at 72°C for 40 seconds. For the following 33 cycles annealing was performed at 60°C. The last extension step at 72°C was extended to 3 minutes. Following amplification, melting curves were acquired during a linear temperature transition from 65 to 90°C with increments of 0.2°C per 10 seconds. Bisulfite primer sets were validated for gene specificity on bisulfite treated methylated CpGenome Universal Methylated Mouse DNA Standard (Millipore, Amsterdam, The Netherlands) and unmethylated WT C57BL/6 mice DNA as reference control sample.

Supplementary Table S1: Primers for methylation assay

Gene	Primer sequence (5'-> 3')	Amplicon size	Number of CpG's	Relative to TSS	Tm unmethylated amplicon	Tm methylated amplicon
Lgr5	GGGTGTTTGGAAAGTAGGGTT<br>CACCGACTTCTACAACTCACCAAAC	268 bp	22	-70 to +199	79.40 °C	82.8 °C
Rosa	GGTGTAATGGTGTGTAAAGGTAGTTG<br>CCTAAATCAAAATAAAAATCCAACAAA	192 bp	8	-684 to -493	78.8 °C	80.6 °C

Supplementary Table S2: Number of tumors stained for EGFP and LacZ

Regimen	# stained for EGFP	# stained for lacZ early injected	# stained for lacZ late injected
Haired chemocarcinogenesis	7	29	11
Hairless chemocarcinogenesis	6	11	15
Hairless UV-carcinogenesis	19	12	16

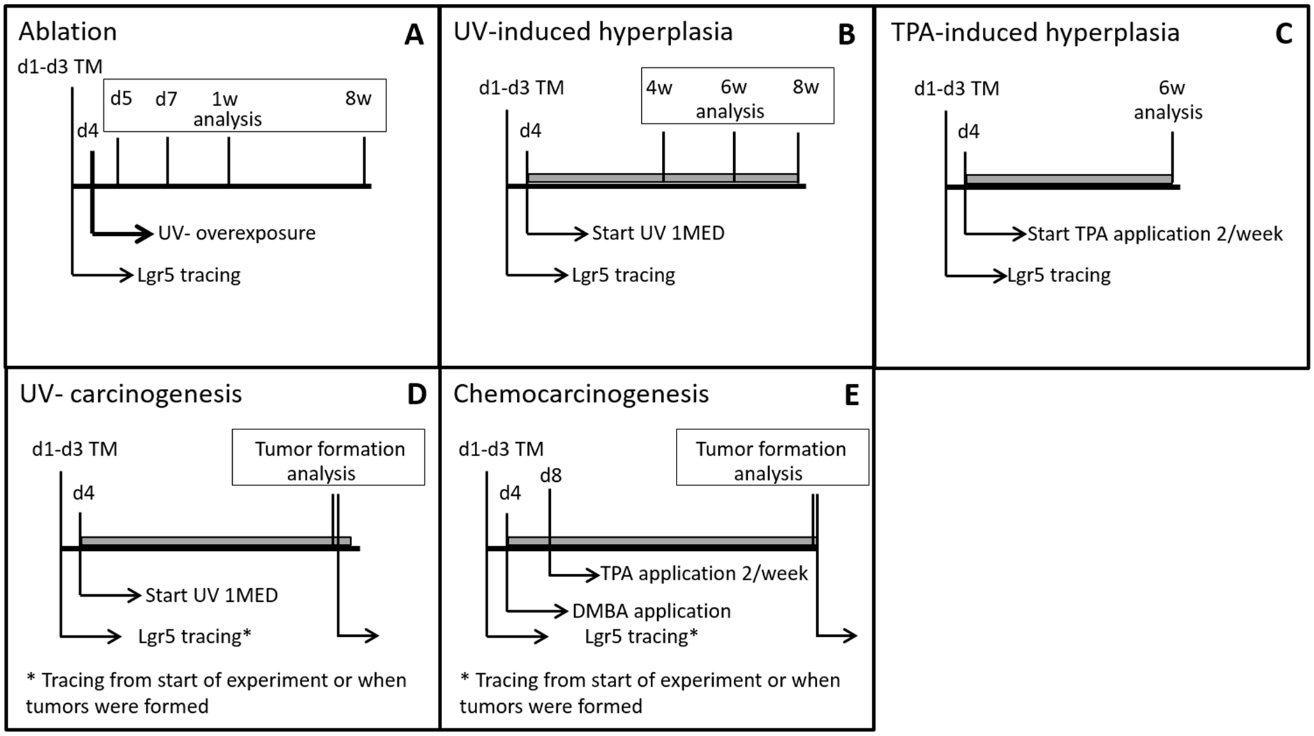

Supplementary Figure S1: Experimental outline. Time line of the experiments including time points of sample collection for analyses. TM= tamoxifen injection, d= day, w=week. In experiments **A, B** and **C** the lineage tracing started at the beginning of the experiment. In experiment **D** and **E** the tracing started either at the start of the experiment or when tumors were formed.

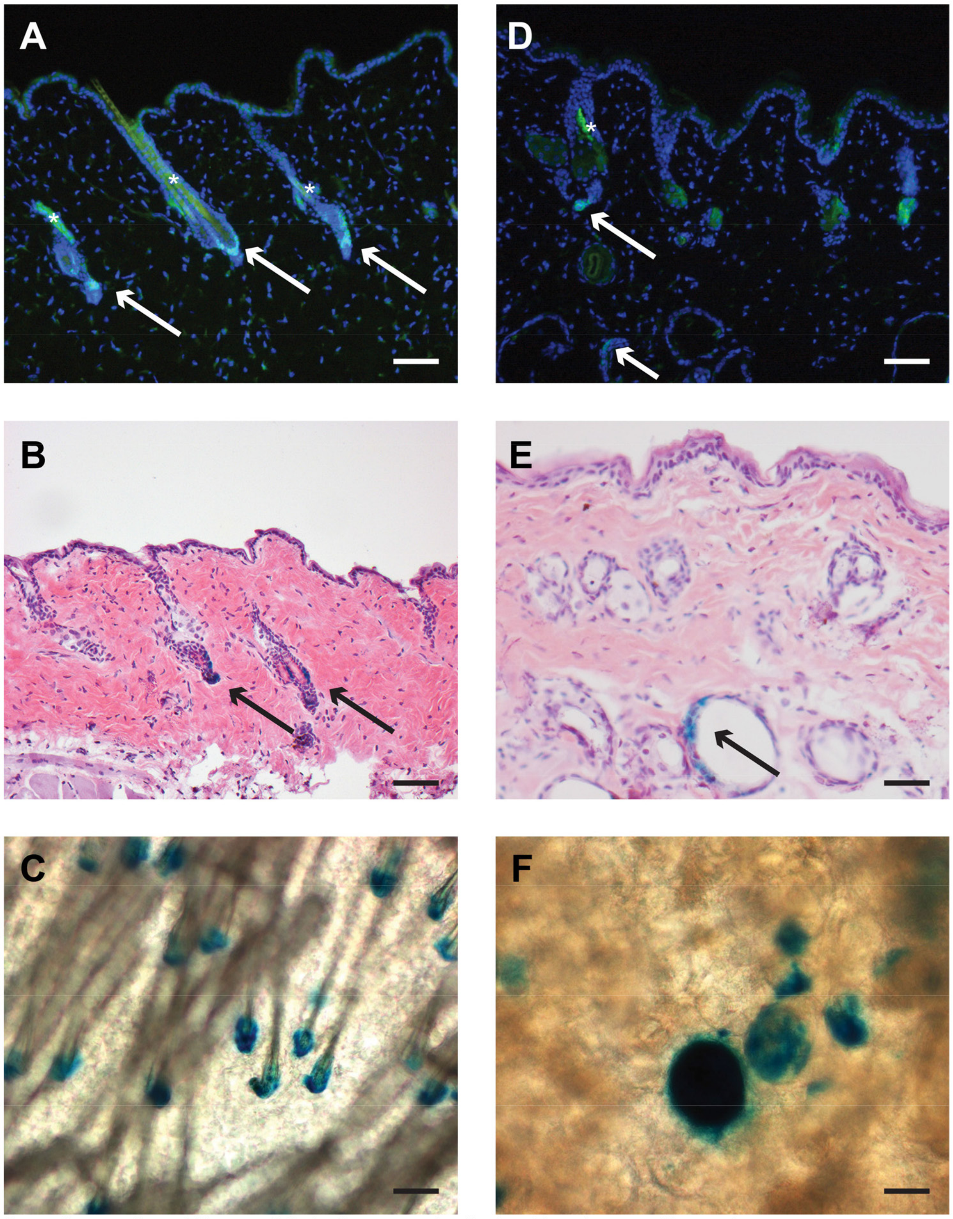

Supplementary Figure S2: In homeostasis Lgr5+ stem cells and progeny are present in the lower part of the hair follicles and in cysts. Skin sections of Lgr5-EGFP-Ires-CreERT2/R26R-LacZ haired **A-C** and hairless **D-F** mice were stained for EGFP (A+D) or LacZ expression (B, E, F after 8 weeks tracing, C after 4 weeks). Skin whole mounts in top view (C+F). EGFP expression representing Lgr5 stem cells was present in the lower part of the hair follicle in haired mice (A, see arrows) and in the hair follicle remnant and cysts of hairless mice (D, see arrows). LacZ lineage tracing shows the Lgr5+ stem cells and their progeny in haired (B+C) and hairless mice (E+F), in the lower part of the hair follicle and in cysts respectively. *= auto fluorescence from hairs. Scale bar in A,B,C,D,F= 100µm, scale bar in E= 50µm

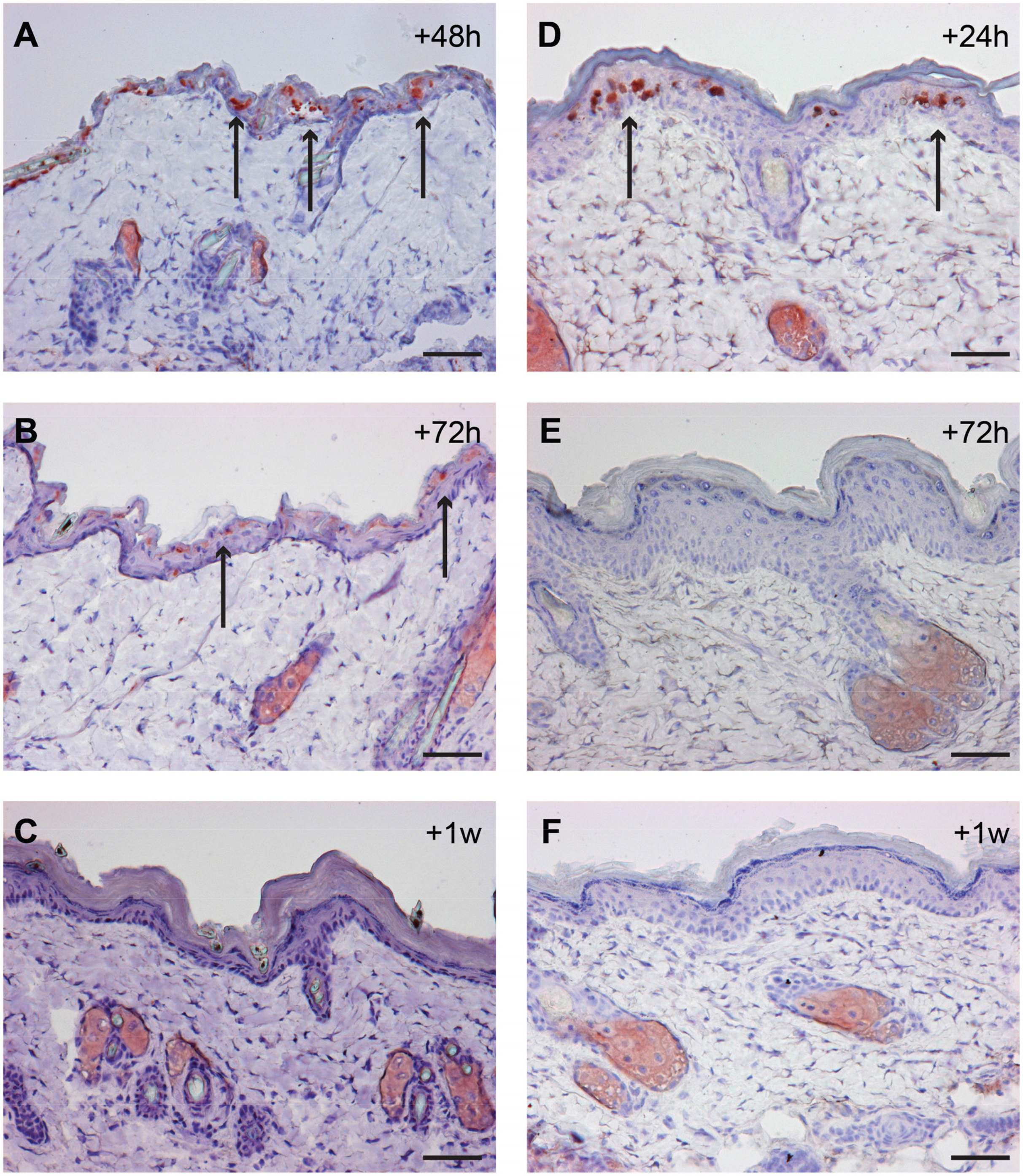

Supplementary Figure S3: Anti-Caspase3 staining showing apoptosis after UV overexposure and largely an ablation of the epidermal basal layer in either haired A-C or hairless mice D-F Anti-Caspase3 staining (red, see arrows) shows apoptotic cells. Pictures show different time points after UV overexposure. In hairless mice the apoptotic cells have already moved out of the epidermis 72h after overexposure (E), but in haired mice they are still present in the upper layer of the epidermis (B). At 1 week after overexposure no apoptotic cells are found anymore in haired or hairless skin (C+F). Scale bars depict 50 um.

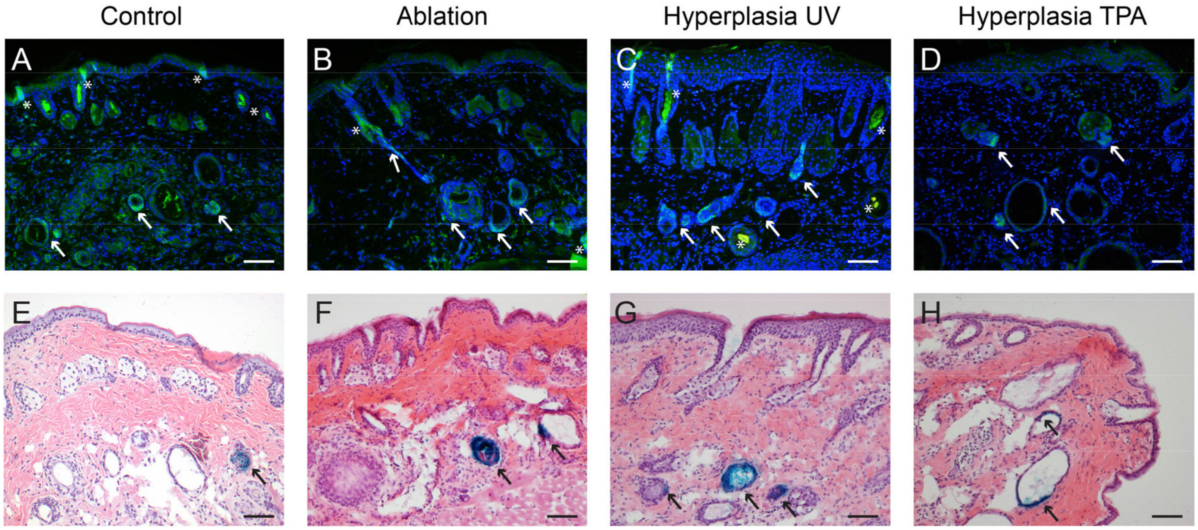

Supplementary Figure S4: Lgr5+ stem cells and their progeny remain in homeostatic positions in hairless mice over 8 weeks of treatment with either UV or TPA. Anti-EGFP staining was used to localize the Lgr5+ stem cells **A-D** and LacZ staining was used to localize their progeny **E-H.** Lgr5+ stem cells and their progeny were found in the same locations after ablation (B+F), hyperplasia induced by UV (C+G) and hyperplasia induced by TPA (D+H); as in homeostasis (shown in the control mice, A+E). Representative pictures of the different experiments and the different time points are shown. scale bar represents 100µm.

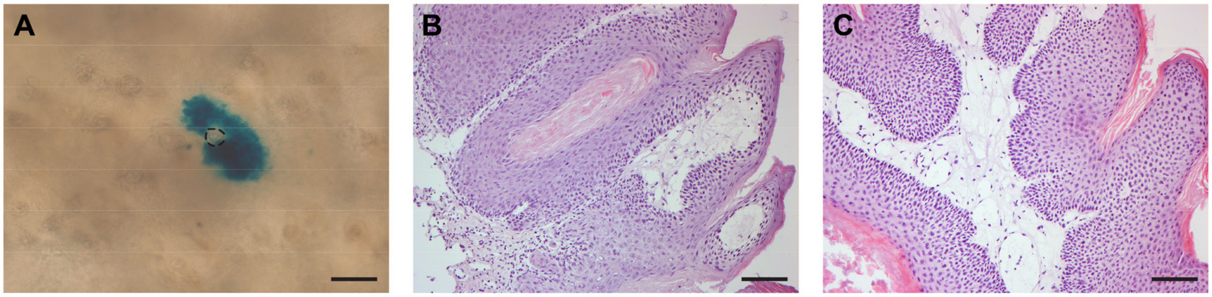

Supplementary Figure S5: Lgr5+ stem cell progeny is present in inter-tumoral skin but absent in chemically induced tumors in hairless mice. Samples of hairless mice subjected to chemocarcinogenesis were stained for LacZ+ cells. Whole mount inter-tumoral skin after chemocarcinogenesis **A** showed islands of Lgr5 progeny in the IFE. Hair follicle orifice contoured (A). Lineage tracing by LacZ activation was induced early, just before tumor initiation **B** or late when tumors were growing out **C.** No Lgr5 progeny was found in these tumors. Scale bar= 100µm.

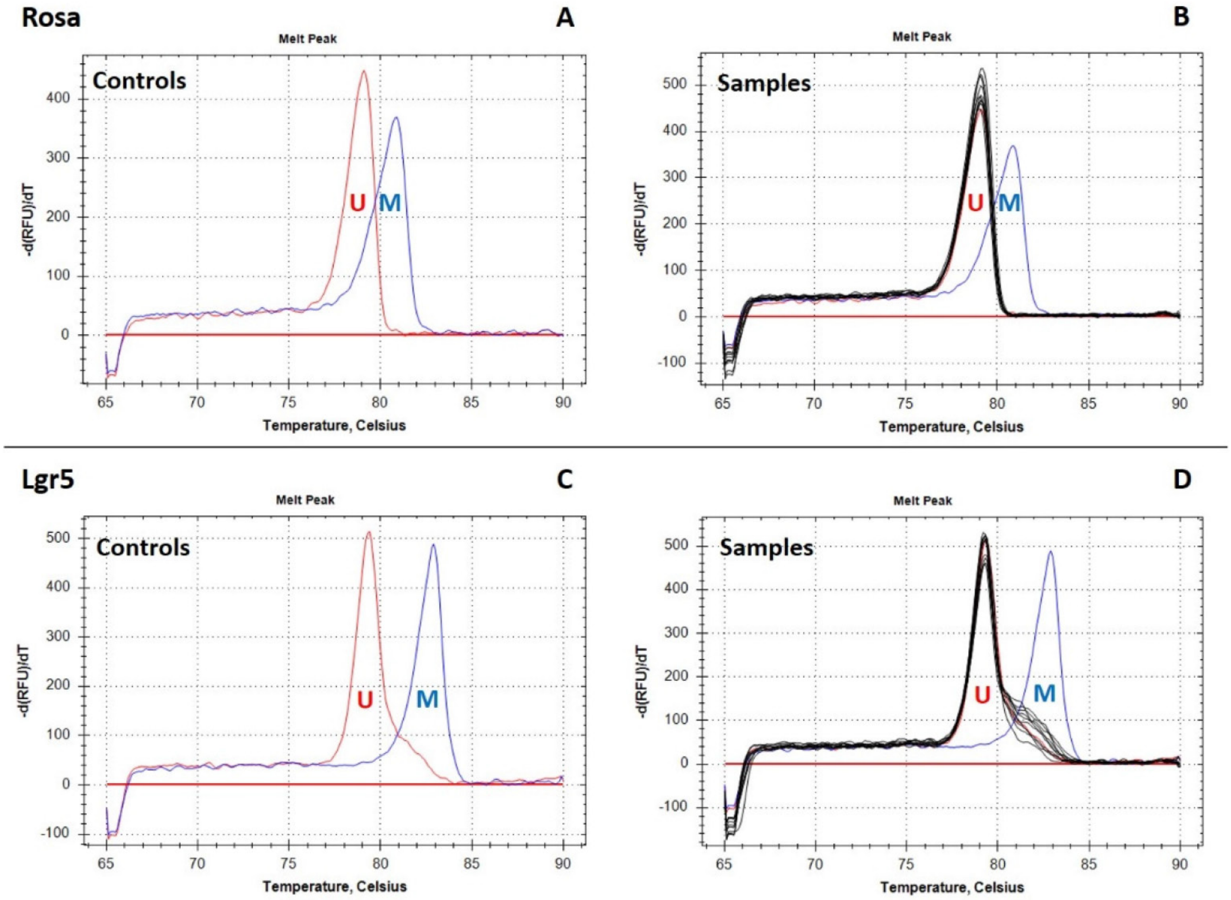

Supplementary Figure S6: Methylation-specific melting curve analysis (MS-MCA) shows absence of methylation of CpG sites in the *Lgr5* and *Rosa* promoter. MS-MCA of control samples showed either completely unmethylated (U) or methylated (M) bisulphite-converted DNA amplified with *Rosa* promoter specific primers **A** or *Lgr5* promoter specific primers **C** (see Supplementary Table SI for primers and their specific melting temperatures). **B** and **D** combined MS-MCA melting curve profiles for 10 samples and controls plotted jointly in one graph, showing a single peak at the Tm for unmethylated DNA amplified with *Rosa* promoter specific primers (B) or *Lgr5* promoter specific primers (D).

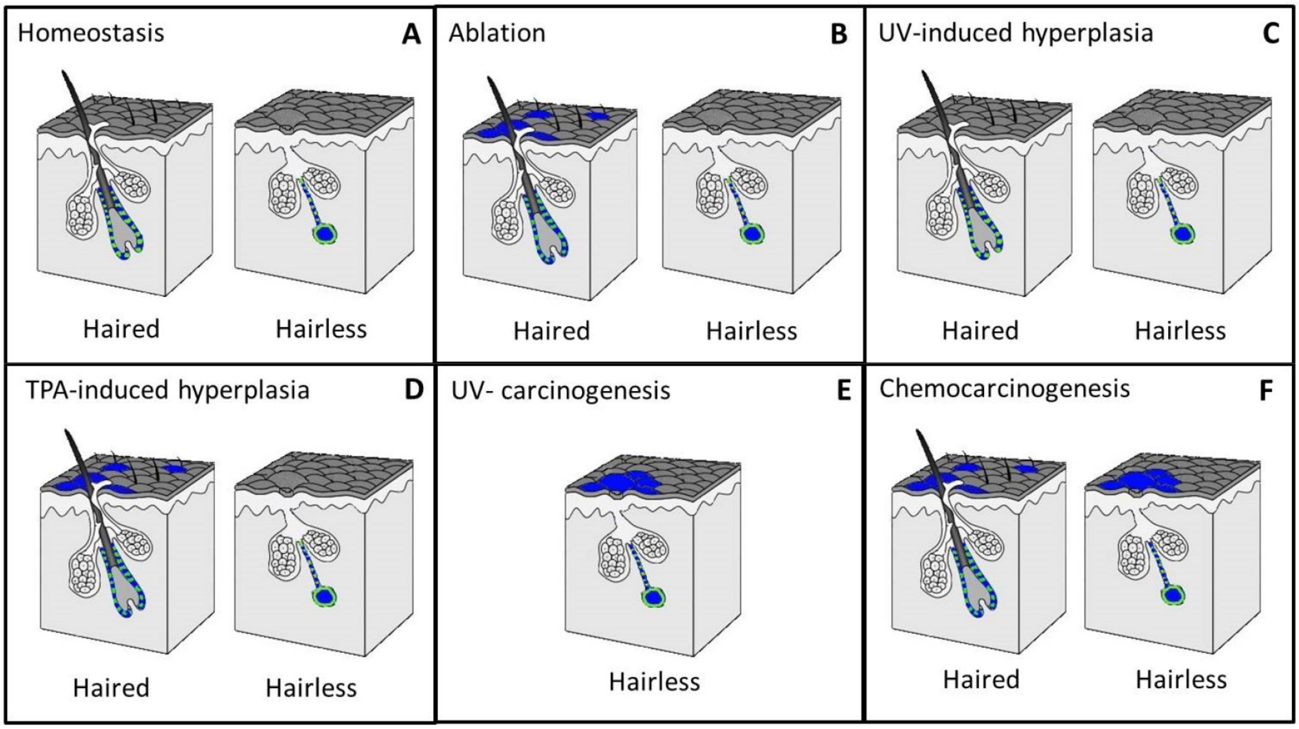

Supplementary Figure S7: Schematic representation of the localization of Lgr5+ stem cells (green) and their progeny (blue) in haired and hairless mice. The stem cells remained at the same location after treatment as during homeostasis **A-F** The progeny of Lgr5+ stem cells was found interfollicularly in haired mice after Ablation (B), TPA-induced hyperplasia (D) and chemocarcinogenesis (F). In hairless mice progeny of Lgr5+ stem cells was only found in the IFE in between the tumors in the carcinogenesis experiments (E+F).
